# Improving feeding and growth of HIV-positive children through nutrition training of frontline health workers in Tanga, Tanzania

**DOI:** 10.1186/s12887-017-0840-x

**Published:** 2017-04-04

**Authors:** Bruno F. Sunguya, Linda B. Mlunde, David P. Urassa, Krishna C. Poudel, Omary S. Ubuguyu, Namala P. Mkopi, Germana H. Leyna, Anna T. Kessy, Keiko Nanishi, Akira Shibanuma, Junko Yasuoka, Masamine Jimba

**Affiliations:** 1 School of Public Health and Social Sciences, Muhimbili University of Health and Allied Sciences, P.O Box 65489, Dar es Salaam, Tanzania; 2 Department of Community and Global Health, Graduate School of Medicine, The University of Tokyo, 7-3-1, Hongo, Bunkyo-ku, Tokyo, 113-0033 Japan; 3 Department of Public Health, School of Public Health and Health Sciences, University of Massachusetts Amherst, Arnold House, 715 North Pleasant St, Amherst, MA 01003-9304 USA; 4 Muhimbili National Hospital, P.O. Box 65000, Dar es Salaam, Tanzania

**Keywords:** Nutrition training, Feeding practices, Nutrition status, HIV/AIDS, Midlevel providers

## Abstract

**Background:**

Nutrition training can boost competence of health workers to improve children’s feeding practices. In this way, child undernutrition can be ameliorated in general populations. However, evidence is lacking on efficacy of such interventions among Human Immunodeficiency Virus (HIV)-positive children. We aimed to examine the efficacy of a nutrition training intervention to improve midlevel providers’ (MLPs) nutrition knowledge and feeding practices and the nutrition statuses of HIV-positive children in Tanga, Tanzania.

**Methods:**

This cluster-randomized controlled trial was conducted in 16 out of 32 care and treatment centers (CTCs) in Tanga. Eight CTCs were assigned to the intervention arm and a total of 16 MLPs received nutrition training and provided nutrition counseling and care to caregivers of HIV-positive children. A total of 776 pairs of HIV-positive children and their caregivers were recruited, of whom 397 were in the intervention arm. Data were analyzed using instrumental variable random effects regression with panel data to examine the efficacy of the intervention on nutrition status through feeding practices.

**Results:**

Mean nutrition knowledge scores were higher post-training compared to pre-training among MLPs (37.1 vs. 23.5, *p* < 0.001). A mean increment weight gain of 300 g was also observed at follow-up compared to baseline among children of the intervention arm. Feeding frequency and dietary diversity improved following the intervention and a 6 months follow-up (*p* < 0.001). An increase in each unit of feeding frequency and dietary diversity were associated with a 0.15-unit and a 0.16-unit respectively decrease in the child underweight (*p* < 0.001).

**Conclusions:**

Nutrition training improved nutrition knowledge among MLPs caring for HIV-positive children attending CTCs in Tanga, Tanzania. Caregivers’ feeding practices also improved, which in turn led to a modest weight gain among HIV-positive children. To sustain weight gain, efforts should be made to also improve households’ food security and caregivers’ education in addition to inservice nutrition trainings. The protocol was registered on 15/02/2013, before the recruitment at *ISRCTN trial registry* with the trial registration number: ISRCTN65346364.

## Background

The global burden of child undernutrition is declining. However, the rates still vary widely among low-income countries [1], with the brunt of the burden of undernutrition still falling on just a few [2]. For example, only 14 countries – all low-income – harbor 80% of the world’s stunted children [2]. Poor feeding practices [3], food insecurity [4], and poverty are important factors behind such undernutrition. These countries also suffer from heavy burdens of Human Immunodeficiency Virus/Acquired Immunodeficiency Syndrome HIV/AIDS and, which further worsens child undernutrition [5].

In low-income countries, undernutrition among HIV-positive children is largely associated with poor feeding practices, low education levels among caregivers, and poverty. Food insecurity is persistent among families of HIV-positive children and is also associated with child undernutrition [4, 6]. However, even in regions with high food production, HIV-positive children are subject to high levels of undernutrition [7, 8]. In such regions, caregivers with poor nutrition knowledge are more likely to feed their children with a low quality and diversity of foods and at a lower frequency than recommended [7, 9]. Improving nutrition knowledge within such contexts may help to ameliorate child undernutrition.

Caregivers’ nutrition knowledge can be improved if they are properly counseled on proper feeding practices based on the local food availability [10]. To achieve this, health workers should first be provided with updated nutrition knowledge, skills, and competence to manage undernutrition. Such skills can be acquired through inservice nutrition training [11, 12]. Nutrition training of health workers has also been effective to improve feeding practices including feeding frequency, dietary diversity, and dietary adequacy [13]. Thus, nutrition counseling by trained health workers has the potential to improve the growth [14] and livelihood of children in the general population [15].

Although evidence is available among children of general populations, evidence on the efficacy of nutrition training and counseling among HIV-positive children remains lacking despite the higher risk of undernutrition and mortality among them. Moreover, typical inservice nutrition training has mostly involved qualified health workers such as qualified nurses, nutritionists, dieticians, clinicians, and other specialized health workers [12, 16–19]. Just a few nutrition-training interventions have also included community health workers and non-medical personnel [20–22]. No study, meanwhile, has reported on any nutrition training being provided to midlevel providers (MLPs).

MLPs constitute the majority of health workers in many developing countries, including Tanzania [23]. Because of health workforce shortages [24], MLPs in Tanzania are left to work in rural and semi-urban areas, where a high number of patients also reside [23]. They receive a 2- to 3-year post-secondary school training to care for simple health conditions [23]. Such training may not be adequate to make them competent to treat complex medical conditions such as severe undernutrition of HIV-positive children with other complications. However, they may be the only available workforce to provide such highly demanding care, with minimal support or trainings in some areas. Therefore, this study had two objectives: first to examine the efficacy of MLPs’ nutrition training to improve their nutrition knowledge, and second to examine the efficacy of such training to improve caregivers’ feeding practices along with the nutrition statuses of affected children.

## Methods

### Study design and area

We conducted this cluster-randomized trial in care and treatment centers (CTCs) in Tanga region, Tanzania. The CTC was taken as the unit of randomization. Detailed information on the CTCs and on the overall health system in this region have been discussed elsewhere [6, 25]. A total of 16 CTCs which fulfilled the selection criteria out of a total 32 in the study area were randomized into intervention and control arms [25], with a total of 8 assigned to each arm using a coin flip randomization method. Pairs of HIV-positive children and their caregivers who attended the selected CTCs were recruited to participate in this study. Two MLPs were selected from each CTC, and only those of the intervention arm received the inservice nutrition training [10]. All participants and their MLPs were blinded with regard to their allocation status. The protocol was registered in February 15^th^ 2013 with a registration number ISRCTN65346364. The recruitment started in July 1^st^ 2013 and ended in July 30^th^ 2014. The manuscript adhered to the CONSORT guidelines.

Undernutrition is rampart among HIV-positive children attending CTCs in this region. In the formative research study preceded the current cluster-randomized controlled trial, about 62% of 748 HIV-positive children recruited among those attending the 9 selected CTCs were stunted [6]. About 39% of them also suffered from low weight for their age. The recruited children had poor feeding frequency and dietary diversity [6]. Reasons proposed for such poor feeding practices included poor caregivers’ nutrition knowledge, food insecurity in families of HIV-positive children, and poverty. Such unprecedented magnitudes of undernutrition were higher compared to the situation among children in the general population in the same region. Prevalence of stunting and underweight were 49.4 and 24.1% respectively among children of the general population in the same region in the year that preceded the current study [26].

### Participants

We recruited three groups of participants in this study: HIV-positive children attending HIV CTCs in the Tanga region; caregivers of such children, who accompany them to the CTCs and supervise their medical and nutritional care at home; and the MLPs who provide nutrition care to the HIV-positive children. The inclusion criteria for HIV-positive children included: children aged 6 months to 14 years, registered at the selected CTCs during baseline phase, and accompanied by his/her caregiver. We excluded children whose caregivers refused to participate, those who lacked a confirmatory HIV test, and those without Antiretroviral therapy (ART) information from the medical data.

We defined a child’s caregiver as a parent or any other adult providing care for the child, accompanying him/her to the clinic [5, 27], and supervising his/her medical and nutritional care. In the intervention arm, nutrition counseling was provided to such caregivers [13].

We selected MLPs based on their roles in HIV-positive children’s routine management and care in their CTCs [25]. We excluded MLPs who did not fit the standard definition [23], including community health workers, home-based caregivers, and other health promotion non-clinical health aides.

Details of sample size calculation were presented in the published research protocol [25]. The minimum calculated sample size was estimated to be 192 pairs of HIV-positive children and their caregivers for each arm (i.e. intervention and control arms). We expanded the sample size to 400 per arm to counteract the effect of loss during follow-up, refusal to continue with the study, children’s attendance without caregivers, and missing data.

### Intervention and follow-up

A total of 16 MLPs in the intervention arm received the 13 h and 40 min nutrition training conducted for two consecutive days in Korogwe district, Tanga. The training was organized into a total of 18 sessions, based on the standard *Guidelines for an Integrated Approach to the Nutritional Care of HIV-infected Children (6 months to 14 years)* produced by the World Health Organization (WHO) [10]. The sessions included theory, practice, and role-playing. Practice sessions involved demonstrations and actual clinical management of undernutrition among HIV-positive children and were carried out in a nearby district hospital. Contents of the training were modified to include risk factors pertinent to the HIV-positive child population in Tanga, feeding practices, and available foods as found in formative research [6].

The trained MLPs provided tailored nutrition counseling and management of undernutrition to HIV-positive children and their caregivers attending monthly to their CTCs. This included assessing nutritional needs, making a nutrition care plan, and providing counseling based on the locally available foods and needed amounts thereof. MLPs also assessed children’s nutritional statuses, and managed undernutrition and other ailments associated with undernutrition. They followed up on observed improvements or deteriorations in feeding practices, weight, and height.

MLPs of the control arm, meanwhile, continued with their standard care for HIV-positive children [5]. This included clinical HIV-staging, adherence counseling, provision of ART, and management of opportunistic infections, similar to MLPs of the intervention arm.

Both intervention and control arms were followed for a period of six months. During the follow-up, we measured feeding practices, nutrition status, and other health-related parameters at the end of the trial and compared them with those observed at baseline.

### Measurements

The outcome variable was nutrition status of HIV-positive children. We intended to measure nutrition status through underweight, wasting, and thinness. To achieve this, we measured weight using the hanging Salter scale® (UK) with minimal clothing for young children. We used Salter digital scale® (Brooklyn, USA) for older children who could stand [28]. We measured height for the 24 months and older children using a Seka® measuring rod [28], and using a marked measuring board in a recumbent position for younger children [29].

We converted the anthropometrics into nutrition indices using the 2006 WHO growth standards [30]. We used the *STATA igrowup* package to convert measurements into weight-for-age z-scores (WAZ-scores), body mass index-for-age z-scores (BMIAZ-scores), and weight-for-height z-scores (WHZ-scores). WAZ was measured for children aged 6 months to 120 months. In this study, a total of 486 children were in this age group. WHZ-score for children aged 6 months to 60 months, of which, only 160 children were eligible for this measure. On the other hand, BMIAZ-scores is used to measure thinness for children up to 14 years of age [31, 32]. This means, all children recruited were eligible for this measure. The z-scores were used as continuous variables for all nutrition statuses to capture the trivial changes. WAZ-score below −2 Standard Deviations (SD) was categorized as underweight. Also, WHZ-score below -2SD categorized as wasting and BMIAZ-score below -2SD categorized as thinness.

We measured feeding practices using feeding frequency and dietary diversity scores. Like in previous studies [5, 6, 27], we asked the caregivers of HIV-positive children about the times they had fed their children in the previous 24 h. We also measured dietary diversity by asking caregivers to provide a list of foods they had fed to their children in the previous 24 h. We made a dietary diversity score based on the list of common foods recalled [6, 25].

We measured nutrition knowledge of MLPs using a standard questionnaire included in the training materials associated with the nutrition training [10]. First, we measured the general knowledge on health- and nutrition-related aspects using scores of the 40 items in all eight sections of the nutrition-training questionnaire. Second, we measured specific aspects of knowledge as follows: three sections (12 items) on general HIV knowledge; one section (4 items) on food preparation knowledge; two sections (8 items) on child feeding practices knowledge; and two sections (8 items) on nutrition counseling skills knowledge. One point was awarded when a participant responded correctly to the given item and zero points were given for a wrong response. For the general knowledge sections, the total scores ranged from 0 to 40. On specific aspects, scores for general HIV knowledge ranged from 0 to 12. For knowledge on food preparation hygiene, scores ranged from 0 to 4. For feeding practices and counseling skills, total scores ranged from 0 to 8 for each. Details of measurements of wealth index, household food insecurity, ART, and HIV clinical stages are found in a protocol paper [25].

### Data collection

Like in our previous studies in Tanzania [5, 6, 27], we used a pretested Swahili questionnaire that was developed in English, translated into Swahili, and then back-translated into English by different researchers to ensure retention of meaning for all variables. Trained research assistants, who were also used in the formative research phase [6], received training on the questionnaire contents, ethics, and anthropometric measurement methods. We conducted face-to-face interviews with the caregivers of HIV-positive children, measured children’s weight and height, and retrieved medical data from their records [25]. Self-administered questionnaires were used to assess the nutrition knowledge of MLPs in the intervention arm before and after the nutrition training.

### Analysis

We analyzed data using both descriptive methods and regression analyses. For descriptive statistics, we used chi-square and t-tests to compare characteristics of participants in the intervention arm and control arm. The compared variables included demographic characteristics, feeding practices, and nutrition status.

We tested the hypotheses using instrumental variable random effects regression analysis [33]. The analysis used panel data to include only the participants who had data at baseline and final follow-up. This two-stage regression analysis first examined whether the nutrition training improved feeding practices (i.e., feeding frequency or dietary diversity) after adjusting for age, sex, caregiver’s education level, household wealth index, and food security.

The second stage of the regression analysis aimed to examine the effect of improved feeding practices. This random effects regressions therefore included either of feeding practices (feeding frequency of dietary diversity) as independent variables and examined changes in nutrition status with two models: underweight and thinness models. The two separate models were built because of the differences between the two outcome variables. In all the models we adjusted for age, sex, education level of the caregiver, household wealth index, and food security. The interaction term of intervention and follow-up was the instrument in this instrument variable random effect model regression. Feeding practices i.e. feeding frequency and dietary diversity was the instrument variable. In this case, feeding frequency or dietary diversity was instrumented by the interaction term of intervention and follow-up. We did not build a wasting-model owing to small number of children with wasting and small sample size of under-five children.

We calculated the effect size of this intervention using Number Needed to Treat (NNT). NNT was calculated while accounting for cluster effects and confounding variables based on the odds ratio (OR) and patient expected event rate (PEER) using the formula from a similar study [34] as follows:

$$ \mathrm{N}\mathrm{N}\mathrm{T} = \left[\left(1\hbox{-} \left(\mathrm{PEER}\ \left(1\hbox{-} \mathrm{OR}\right)\right)\right)\ /\right[\left(1\hbox{-} \mathrm{PEER}\right)\ \mathrm{PEER}\ \left(1\hbox{-} \mathrm{OR}\right)\Big] $$

OR was calculated using logistic regression models with random effects for clusters and adjusted for age, sex, caregiver’s education, wealth index, and food insecurity. PEER was estimated using the event rate of the control group. We used the intention-to-treat principle to analyze the data and set the statistical significance at *p* < 0.05. All analyses were conducted using STATA version 12 (StataCorp, College Station, Texas, USA).

### Ethical considerations

We obtained informed written consent from the training participants and caregivers of HIV-positive children before data collection. This study was approved by the Research Ethics Committee of the University of Tokyo, and the Expedited Review Sub-committee of the Senate Research and Publication Directorate of the Muhimbili University of Health and Allied Sciences.

## Results

### General characteristics of intervention and control arms

**Fig. 1:**
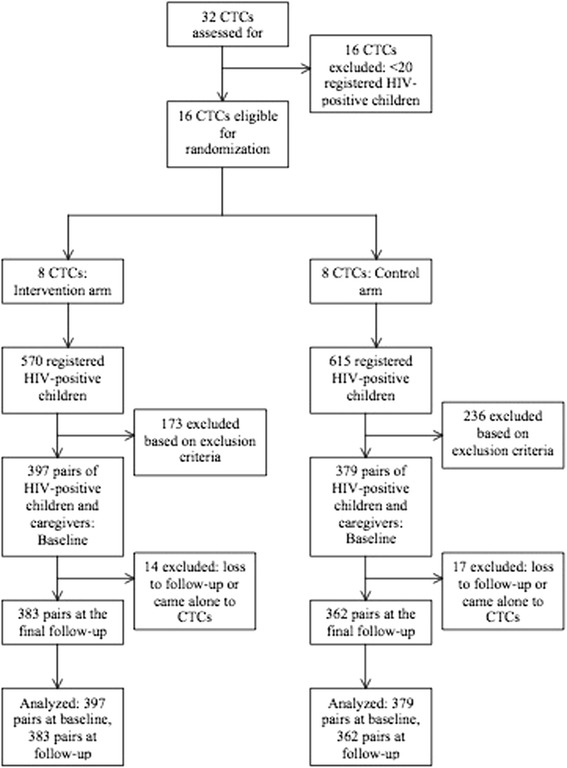
Trial flow chart

Data from 776 pairs of HIV-positive children and their caregivers were available for analysis at baseline. Among them, 397 pairs belonged to the CTCs of the intervention arm (Fig. 1). At the final follow-up, data from 745 pairs were available. Among them, 383 pairs belonged to the intervention arm.

Table 1 shows the general characteristics of participants and compares intervention and control arms. Majority of HIV-positive children (59.4% for the intervention and 64.5% for the control arm) had lost one or both parents. Majority of children in this study were on ART (86.4% in the intervention and 88.9% in the control arm). The mean ART duration for the intervention arm was 36.9 months compared to 33.5 months among those in the control arm. However, 72.8% of the HIV-positive children in the intervention arm and 72.2% of those in the control arm had advanced HIV clinical stages. Finally, 68.3% and 72.6% of children in the intervention and control arms, respectively, lived in households with food insecurity.

**Table 1:** Descriptive characteristics of intervention and control arms

Variable	Total	Intervention			Control			*P*
		n	% (mean)	SD	n	% (mean)	SD	
Age (months)^a^	776	397	(103.6)	43.5	379	(98.2)	45.1	0.097
Sex^b^								
Male	372	199	50.3		173	45.7		0.199
Female	403	197	49.7		206	54.3		
Orphan-hood^b^								
Both parents alive	280	154	40.6		126	35.5		0.388
Only mother alive	166	82	21.6		84	23.7		
Only father alive	117	54	14.3		63	17.7		
Both parents dead	171	89	23.5		82	23.1		
HIV-clinical stage^b^								
Stage I	52	23	5.9		29	8.0		0.054
Stage II	155	83	21.3		72	19.8		
Stage III	463	230	59.1		233	64.2		
Stage IV	82	53	13.7		29	8.0		
On ART^b^								
No	96	54	13.6		42	11.1		0.293
Yes	679	343	86.4		336	88.9		
ART duration^a^								
Mean months	677	357	(36.9)	27.8	320	(33.5)	27.5	0.108
Caregiver^b^								
Mother	352	179	45.1		173	45.7		0.876
Other	424	218	54.9		206	54.3		
Education level (caregiver)^b^								
Not formal	208	113	28.5		95	25.1		0.074
Primary	497	241	60.7		256	67.7		
Secondary &above	70	43	10.8		27	7.2		
Wealth index^b^								
Lowest	159	108	27.2		51	13.5		<0.001
Low	152	55	13.9		97	25.6		
Middle	156	61	15.4		95	25.1		
High	154	76	19.1		78	20.5		
Highest	155	97	24.4		58	15.3		
Food security (HFIAS)^b^								
Food-secure	230	126	31.7		104	27.4		0.190
Food-insecure	546	271	68.3		275	72.6		

^a^ *t*-test; ^b^Chi-square test

### Effectiveness of nutrition training in improving nutrition knowledge

Table 2 shows the effect of nutrition training on MLPs knowledge, including nutrition knowledge aspects. The knowledge score of MLPs improved after the training compared to the pre-training test (37.1 vs. 23.5, *p* < 0.001). Moreover, all four main aspects of knowledge scores tested in this study improved significantly at the post-training test compared to the pre-training test session. For example, the mean value for MLPs’ knowledge score on pediatric HIV/AIDS improved from 9.8 to 14.5 (*p* < 0.001); knowledge on food preparation hygiene improved from 2.9 to 4.6 (*p* < 0.001); knowledge on feeding practices improved from 4.4 to 9.3 (*p* < 0.001); and knowledge on nutrition and feeding counseling improved from 6.4 to 8.8 (*p* < 0.001) after the training.

**Table 2:** MLPs’ nutritional knowledge before and after receiving nutrition training for HIV-positive children in the intervention arm

Aspect of knowledge	N	Mean	SD	*P*
Total knowledge score				
Pre-training	16	23.5	6.5	<0.001
Post-training	16	37.1	3.1	
Pediatric HIV/AIDS				
Pre-training	16	9.8	0.9	<0.001
Post-training	16	14.5	0.2	
Food preparation hygiene				
Pre-training	16	2.9	1.0	<0.001
Post-training	16	4.6	1.0	
Feeding practices				
Pre-training	16	4.4	2.1	<0.001
Post-training	16	9.3	0.9	
Nutrition counseling				
Pre-training	16	6.4	1.6	<0.001
Post-training	16	8.8	1.7	

### Changes in feeding practices

Table 3 shows the changes in feeding practices following the intervention and at final follow-up. HIV-positive children in the intervention arm had a slightly higher mean feeding frequency at baseline compared to those of the control arm (2.8 vs. 2.6, *p* = 0.041). However, a significant increase in feeding frequency was observed in the intervention arm compared to the control arm at the final follow-up (4.4 vs. 3.1, *p* < 0.001). To achieve the WHO’s stipulated feeding frequency of 5 times a day, the Number Needed to Treat (NNT) to change one child’s feeding frequency was 12.1.

**Table 3:** Changes of feeding practices, anthropometry, and nutrition status between intervention and control arms

Variable	Total	Intervention arm			Control arm			*P*
		n	Mean (%)	SD	n	Mean (%)	SD	
Total feeding frequency^a^								
Baseline	776	397	2.8	0.8	379	2.6	0.6	0.041
Month 6	745	383	4.4	0.7	362	3.1	0.8	<0.001
Feeding frequency above 5^b^								
Baseline	776	12	(3.1)	-	5	(1.3)	-	0.105
Month 6	745	172	(44.9)	-	34	(9.4)	-	<0.001
Total dietary diversity score^a^								
Baseline	776	397	2.8	0.7	379	2.9	0.9	0.061
Month 6	745	383	4.3	0.8	362	3.4	0.7	<0.001
Dietary diversity at least 3/day^b^								
Baseline	776	276	(69.5)	-	259	(68.3)	-	0.772
Month 6	745	379	(99.0)	-	336	(92.8)	-	<0.001
Weight (kg)^a^								
Baseline	776	337	21.7	7.4	379	20.9	7.9	0.134
Month 6	745	383	22.0	7.1	362	20.5	7.4	0.003
Weight-for-age z-scores^a^								
Baseline	486	238	−1.5	1.3	248	−1.6	1.5	0.229
Month 6	472	243	−1.1	1.3	228	−1.9	1.3	<0.001
Weight-for-height z-scores^a^								
Baseline	160	80	0.4	1.8	80	−0.6	1.9	0.001
Month 6	141	72	1.6	2.3	69	−0.4	1.3	<0.001
BMI-for-age z-scores^a^								
Baseline	774	396	−0.5	1.6	378	−0.8	1.8	0.011
Month 6	745	383	0.2	2.1	362	−0.9	1.6	<0.001
Underweight (age 6-120months)^b^								
Baseline	487	79	(33.2)	-	105	(42.2)	-	0.041
Month 6	471	55	(22.6)	-	104	(45.6)	-	<0.001
Thinness (age 6 months-14 years)^b^								
Baseline	776	59	(14.9)	-	69	(18.2)	-	0.210
Month 6	745	46	(12.0)	-	71	(19.6)	-	0.004

^a^ *t*-test; ^b^Chi-square test

Number Needed to Treat (NNT)

Feeding frequency above 5/day = 12.1; Dietary diversity at least 3/day = 1.4; Underweight = 3.9; Thinness =7.0; Stunting = 40.7

Similarly, dietary diversity improved significantly from 2.8 and 2.9 among the children of the intervention and control arms, respectively for baseline scores to 4.3 compared to 3.4 in the intervention and control arm respectively for follow up scores.

### Changes in anthropometry among HIV-positive children

Weight was not significantly different at baseline between the HIV-positive children attending CTC intervention and control arms (Table 3). However, at the final follow-up, mean weight increased significantly within the intervention arm compared to baseline, and was significantly higher compared to that of the control arm (22.0 kg vs. 20.5 kg, *p* = 0.003). A mean increment weight gain of 300 g was also observed at follow-up compared to baseline among children of the intervention arm. Changes in weight led to changes in weight-related nutritional indices (WAZ-scores, WHZ-scores, and BMIAZ-scores). The Number needed to treat (NNT) to change underweight status for one child was 3.9.

### Effectiveness of the intervention in improving nutrition status through changes in feeding frequency

Table 4 shows the results of the 2-stage instrumental variable random effects regression. In the first stage regression, feeding frequency increased significantly in the intervention arm and at the 6 months final follow-up compared with the baseline in all the three models as follows: underweight-model: β = 1.15, *p* < 0.001and thinness-model: β = 1.19, *p* < 0.001). After adjusting for important confounders and covariates, feeding frequency generally improved at the final follow-up compared to the baseline (underweight-model: β = 0.39, *p* < 0.001 and thinness-model: β = 0.41, *p* < 0.001).

**Table 4:** Effect of the intervention on nutrition status through changes in feeding frequency: Instrumental variable random effects regression

Variable	Underweight-model			Thinness-model		
	β	95% CI	*P*	β	95% CI	*P*
First stage: Changes in feeding frequency at 6 months post-intervention						
Intervention*follow-up	1.15	0.98, 1.31	<0.001	1.19	1.08, 1.30	<0.001
Intervention	0.12	−0.03, 0.26	0.129	0.12	−0.07, 0.31	0.226
Follow-up	0.39	0.27, 0.51	<0.001	0.41	0.33, 0.49	<0.001
Age	−0.01	−0.01, 0.01	0.849	−0.01	−0.01, 0.01	0.225
Sex	−0.06	−0.18, 0.06	0.333	0.11	−0.02, 0.25	0.111
Caregiver’s education	0.02	−0.08, 0.12	0.671	−0.01	−0.09, 0.08	0.918
Wealth index	0.04	−0.01, 0.09	0.100	0.01	−0.01, 0.08	0.079
Food insecurity	0.01	−0.01, 0.01	0.892	−0.03	−0.01, 0.01	0.380
Second stage: random effects regression: changes in nutrition status as a result of changes in feeding frequency						
Feeding frequency	−0.15	−0.24, −0.07	<0.001	−0.04	−0.08, 0.01	0.059
Intervention	−0.07	−0.16, 0.02	0.133	−0.03	−0.11, 0.05	0.402
Follow-up	0.12	0.03, 0.21	0.012	0.01	−0.03, 0.06	0.541
Age	0.01	0.01, 0.01	0.018	0.01	0.01, 0.01	<0.001
Sex	−0.02	−0.09, 0.05	0.575	−0.01	−0.06, 0.05	0.832
Caregiver’s education	−0.01	−0.06, 0.05	0.917	−0.02	−0.06, 0.01	0.204
Wealth index	−0.02	−0.05, 0.01	0.083	−0.01	−0.02, 0.01	0.691
Food insecurity	0.01	−0.01, 0.01	0.514	−0.01	−0.01, 0.01	0.154

Intervention*follow-up = interaction term between intervention and follow-up

Intervention: subjects at the intervention compared to control arm

Follow-up: subjects at the follow-up compared to the baseline

In the second stage, an increase in each unit of feeding frequency was associated with a 0.15-unit decrease in the child underweight (*p* < 0.001). Caregiver’s education and food insecurity were also associated with child undernutrition.

### Effectiveness of the intervention in improving nutrition status through changes in dietary diversity

After adjusting for important confounders and covariates, feeding frequency generally improved at the final follow-up compared to the baseline (underweight-model: β = 0.48, *p* < 0.001and thinness-model: β = 0.46, *p* < 0.001) (Table 5). In the second stage, an increase in one unit of dietary diversity was associated with a 0.16-unit decrease in the child underweight (*p* < 0.001) but not thinness (*p* = 0.078). Other factors associated with undernutrition included age, wealth index and food insecurity.

**Table 5:** Effect of the intervention on nutrition status through changes in dietary diversity: Instrumental variable random effect regression

Variable	Underweight-model			Thinness-model		
	β	95% CI	*P*	β	95% CI	*P*
Intervention*follow-up	1.11	0.94, 1.28	<0.001	1.10	0.96, 1.24	<0.001
Intervention	−0.08	−0.22, 0.07	0.310	−0.07	−0.19, 0.05	0.254
Follow-up	0.48	0.35, 0.60	<0.001	0.46	0.36, 0.56	<0.001
Age	0.01	−0.01, 0.01	0.911	−0.01	−0.01, 0.01	0.349
Sex	−0.06	−0.17, 0.06	0.354	0.01	−0.10, 0.10	0.973
Caregiver’s education	0.03	−0.07, 0.13	0.544	0.02	−0.06, 0.10	0.629
Wealth index	0.01	−0.04, 0.06	0.583	0.01	−0.03, 0.04	0.865
Food insecurity	−0.01	−0.02, −0.01	0.001	−0.01	−0.02, −0.01	<0.001
Second stage: random effects regression: changes in nutrition status as a result of changes in dietary diversity						
Variable	Underweight (WAZ < −2SD)			Thinness (BMIAZ < −2SD)		
	β	95% CI	*P*	β	95% CI	*P*
Dietary diversity	−0.16	−0.25,-0.07	0.001	−0.05	−0.10, 0.01	0.078
Intervention	−0.10	−0.18,-0.01	0.022	−0.04	−0.09, 0.01	0.121
Follow-up	0.13	0.03, 0.24	0.015	0.03	−0.04, 0.09	0.408
Age	0.01	0.01, 0.01	0.021	0.01	0.01, 0.01	<0.001
Sex	−0.02	−0.09, 0.05	0.614	−0.01	−0.05, 0.04	0.887
Caregiver’s education	0.01	−0.06, 0.21	0.934	−0.03	−0.07, 0.01	0.073
Wealth index	−0.03	−0.06, 0.06	0.047	−0.01	−0.03, 0.01	0.108
Food insecurity	−0.01	−0.01, 0.01	0.684	−0.01	−0.01,-0.01	0.022

Intervention*follow-up = interaction term between intervention and follow-up

Intervention: subjects at the intervention compared to control arm

Follow-up: subjects at the follow-up compared to the baseline

## Discussion

This is the first cluster-randomized controlled trial to examine the efficacy of nutrition training in improving MLPs’ nutrition education. It also serves as the first study to examine the efficacy of such training using standard WHO guidelines [10] and local determinants of undernutrition [6] toward improving feeding practices and nutrition status among HIV-positive children. In this study, nutrition training of MLPs improved their nutrition knowledge. It also improved feeding frequency and dietary diversity among HIV-positive children at the 6-month follow-up in the intervention arm. As a result, a small but significant weight gain and related improvements in nutrition statuses were detected among HIV-positive children of the intervention arm.

The following three pathways may help explain such gains. First, the nutrition training improved the nutrition knowledge of MLPs in the intervention arm. In Tanga, MLPs who care for HIV-positive children, had a low level of nutrition knowledge before the training [6]. However, through this intervention, they could improve their knowledge significantly and thus exert positive influences on the caregivers as explained below.

Second, the feeding practices improved significantly among HIV-positive children in the intervention arm. In the formative research [6], 88.1% of 748 children had a feeding frequency lower than that recommended by the WHO for HIV-positive children [10]. About 62% of them also had low levels of dietary diversity. These factors were positively associated with poor nutrition statuses [6]. The mean feeding frequency and dietary diversity increased more in the intervention than the control arm. Even after adjusting for other variables, the intervention arm at follow-up had a significantly higher feeding frequency and dietary diversity. Therefore, nutrition training coupled with nutrition counseling improved feeding practices to a level similar to that observed in general and HIV-negative populations [13]. In this study, the trained MLPs could thus help to transmit nutrition knowledge to caregivers [11, 15, 35], and used available resources to improve feeding practices for their children [15, 36].

Third, the improvement of feeding practices brought a modest weight gain among the observed HIV-positive children in the intervention arm. After adjusting for potential differences between and within groups, improved feeding practices were associated with better nutrition statuses. Higher feeding frequency is known to increase the amount of food absorbed and replenishes losses sustained through catabolic processes triggered by HIV and opportunistic infections [10, 37–39]. Increased dietary diversity also improves appetite and thereby increases the amount of food consumed by a child, even apart from the added nutritional value [10, 39]. The concomitant increases in both factors must have contributed to the observed weight gain among children in the intervention arm.

Despite using a randomized controlled design, our study was not free of limitations. First, we depended on self-report and the recall of caregivers in measuring feeding practices. However, the current findings at baseline were not significantly different from those identified in the formative research [6]. Second, we lost a total of 31 pairs of HIV-positive children and their caregivers in our final analyses, as we could not interview children who came alone to the CTCs unaccompanied by their caregivers [25]. However, such children were almost evenly distributed in both arms. Third, a 6-month follow-up may be too short to observe significant changes in long-term outcome variables such stunting. Therefore, we could not see the impact of this intervention in stunting that need a relatively longer follow-up time, an avenue for future studies. Fourth, some questionnaire had missing data on some variables. This led into small differences in total values in some demographic characteristics. We did not exclude questionnaires with missing variables unless they also had missing information on outcome variables. Fifth, we cannot ascertain any changes in the nutrition knowledge for caregiver, as we did not measure it before, during, and after the intervention. This is an important area for future research. Despite its limitations, this is the first study to assess the efficacy of nutrition training toward improving MLPs’ feeding practices and, thereby, the nutrition status of HIV-positive children.

## Conclusions

In conclusion, this study found out that, providing nutrition training to MLPs effectively improved their nutrition knowledge, which in turn improved feeding practices among HIV-positive children in Tanga region, Tanzania. The improved feeding practices brought about a small weight gain in this food-secure region. Even where the health workforce is limited, providing nutrition training to the available workforce can help to ameliorate undernutrition among HIV-positive children. Nutrition training alone, however, may not be enough to ameliorate growth faltering. Efforts are thus needed to improve food insecurity, poverty, and education levels among the caregivers of HIV-positive children toward bringing about lasting and sustainable improvements in nutrition.

## Abbreviations

ART: Antiretroviral therapy
BMIAZ: Body Mass Index-for-Age Z-score
CI: Confidence interval
CTC: Care and treatment center
CTC: Care and treatment center
HIV: Human immunodeficiency virus
MLP: Mid level provider
NNT: Number Needed to Treat
OR: Odds ratio
PEER: Patient expected event rate
SD: Standard Deviation
UK: United Kingdoms
USA: United States of America
WAZ: Weight-for-Age Z-score
WHO: World Health Organization
WHZ: Weight-for-Height Z-score.
